# A new continental hydrogen play in Damara Belt (Namibia)

**DOI:** 10.1038/s41598-024-62538-6

**Published:** 2024-05-22

**Authors:** V. Roche, U. Geymond, M. Boka-Mene, N. Delcourt, E. Portier, S. Revillon, I. Moretti

**Affiliations:** 1 Laboratoire des Fluides Complexes et leurs Réservoirs - IPRA, E2S-UPPA, TotalEnergies, CNRS, Université de Pau et des Pays de l’Adour, UMR5150, Pau, France; 2 Laboratoire de Planétologie et Géosciences, LPG UMR 6112, CNRS, Le Mans Université, Univ Angers, Nantes Université, Avenue Olivier Messiaen, 72085 Le Mans, France; 3 Institut de physique du globe de Paris, CNRS, Université Paris Cité, Paris, France; 4 45-8 Energy, Lyon, France; 5 SEDISOR/-Geo-Ocean, Univ Brest, CNRS, Ifremer, UMR6538, Plouzané, France

**Keywords:** Structural geology, Petrology, Geochemistry

## Abstract

Serpentinization is commonly presented as the main source of natural hydrogen (H_2_) in the continental domains. However, recent works in Australia and Brazil showed that Archean–Paleoproterozoic banded iron formations could be another natural source of H_2_ gas. Although the reaction that produces hydrogen is similar (Fe^2+^ oxidation—H_2_O reduction process), the iron content may be higher in banded iron formations than in mafic igneous lithologies, potentially generating H_2_ more efficiently. Here, we present structural evidence that reported H_2_ emissions from Waterberg Basin, Namibia are associated with underlying Neoproterozoic banded iron formations—the Chuos Formation. Magnetite, a known H_2_-generating mineral, is ubiquitous and accompanied by other suspected H_2_-generating minerals (biotite and siderite) in Chuos Formation. Magnetite occurs either as pervasive cm to dm continuous metamorphic laminations in foliation and fractures planes and/or diffusely disseminated in metachert and metacarbonate levels. From this, we infer that metamorphism does not negatively affect the Fe^2+^ content that is required to generate hydrogen. H_2_ seepages in Waterberg Basin suggest that an active H_2_-generating system may exist at depth and that the presence of potential traps and reservoirs is likely based on field observations.

## Introduction

The energy transition, including the hydrogen economy, requires better use and understanding of natural carbon-free energy carriers and resources. Typically, hydrogen is an energy-carrier that is manufactured from other energy resources (methane—blue; hydro-electricity—green; coal—grey). The fortuitous Hydroma company discovery in Mali demonstrates that naturally occurring hydrogen (H_2_) is a carbon-free energy resource generated and accumulated underground in continental domains^1,2^. This discovery stimulated H_2_ exploration globally by private companies. However, the dynamics of H_2_ systems, from H_2_-generating rocks to accumulation in reservoirs and their economic viability, remains poorly described and understood. Understanding H_2_ systems is therefore crucial for the development of this new primary carbon-free energy resource that is considered one of the most promising pathways towards a carbon-free energy transition (e.g.^3,4^).

Various geochemical mechanisms (e.g. redox reactions, radiolysis of H_2_O, organic maturation, deep-seated H_2_—e.g.^4,5^) are invoked to explain H_2_ generation in the continental domain. The main challenge is to identify the main process that generates H_2_ at depth. In Namibia, natural H_2_ occurrences were reported recently in Waterberg Basin^6^. Concentrations of gaseous H_2_ were measured in sub-circular topographic depressions (SCDs), similar to gas seeps observed elsewhere (e.g.^7,8^). The presence of Neoproterozoic Chuos Formation banded iron formations close to these gas seeps indicates that redox processes could be the primary drivers of H_2_ generation in this case^6^. Here, the alteration of magnetite into maghemite/hematite by water circulation within the banded iron formations could result in H_2_ generation. This hypothesis is supported by previous studies. Geymond et al.^9^ showed the spatial correlation between banded iron formations and SCDs in Australia, Brazil and South Africa. Based on the drill cores study, they observed that banded iron formations are altered by water close to the surface. They therefore concluded that H_2_ may be generated during banded iron formation weathering. In addition, a recent experimental alteration study demonstrated that a synthetic powder of magnetite generates H_2_ at 80 °C and 200 °C during its alteration^10^. Paradoxically, Neoproterozoic banded iron formations are assumed to contain very low amounts of Fe^2+^ in the literature (e.g.^11,12^), which sparks an ongoing debate regarding the connection between H_2_ generation and iron oxidation^10^. In that case, other mechanisms could be considered such as radiolysis of H_2_O and/or deep-seated H_2_ generation.

### Geological background of the Damara Belt

**Figure 1:**
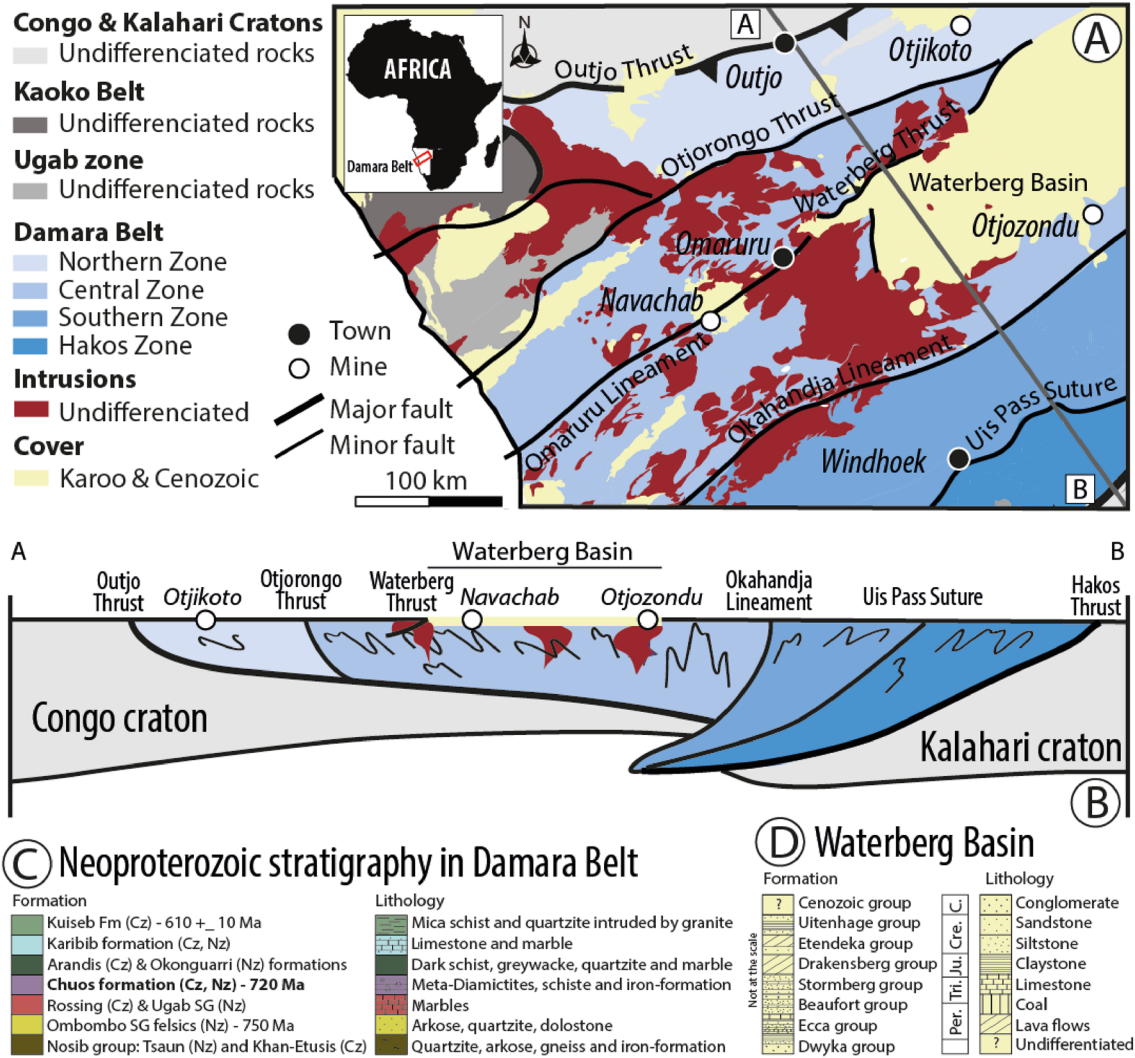
Geological background of Waterberg Basin. (**A**) Simplified geological map of northern Namibia including Damara Belt (after Ref.^13^). The grey indicates the position of the cross-section in (**B**). (**B**) Cross-section modified from Ref.^13^. (**C**) Stratigraphy and sedimentary log in Damara Belt and (**D**) in Waterberg Basin. Note that the legends in (**C**) and (**D**) do not correspond to the map and profile shown in A and B.

Located in central Namibia, between the Kalahari and Congo cratons, Damara Belt trends NE-SW (Fig. 1A) and encompasses a diachronous tectono-metamorphic evolution during its deformation (e.g.^13,14^). Separated by major tectonic contacts, four zones have been identified (Fig. 1A and B). From north to south, these include, Northern, Central, and Southern zones, and Hakos Zone. Central Zone rocks were intruded by several granitic rocks over a long period (568–500 Ma; e.g.^15–17^) whereas Southern Zone was mainly intruded by a large batholith at 508–504 Ma^17,18^. Interestingly, detrital zircons from the Northern Zone to the Central Zone yield similar ages (ranging from 780 to 600 Ma), implying that lithologies are contemporaneous in a large part of Damara Belt (e.g.^19^). These Neoproterozoic rocks are mainly composed of interbedded schists, carbonates, and sandstones (Fig. 1C). Some formations are enriched in iron, such as the Chuos Formation (bold type in Fig. 1C). The sedimentology and stratigraphy of the Chuos Formation is detailed in Ref.^20^ and more recently in several studies (e.g.^21,22^). This formation is found throughout the Damaran Orogeny^20^ and shows various facies revealing different depositional controls along the shelf platform of the southern Central Zone^23^. Chuos Formation consists mainly of diamictite of dam thickness interbedded with cm to dm beds of finely laminated banded ironstones (up to 60 wt% Fe;^22^). This succession is inferred deposited in an ice-proximal to sub-ice glaciomarine shelf environment (e.g.^21,24^) with the interbedded ironstones attributed to a microbial origin for some iron oxides^21^. Chuos Formation thickness is highly variable ranging, from 76 to 1660 m at the north end of the Outjo Thrust^25^, and from a few meters to 200 m in the Northern Zone where the Chuos Formation is metamorphosed^22^.

The sedimentary units forming Waterberg Basin (Fig. 1D) covered all the previous metamorphic units, including the metamorphosed Chuos Formation (Fig. 1C). A structural analysis on the northern side of Waterberg Basin reveals that a set of extensional faults developed first between two major regional lineaments (Otjorongo Thrust and Omaruru lineament, Fig. 1A and B) during the Permo-Triassic^26^. Subsequently, these extensional structures were reactivated as thrust faults juxtaposing the basement onto the Permo-Triassic and Lower Jurassic Karoo clastic sediments that were folded locally during Triassic and Cretaceous times^26^. These sediments belong to Dwyka, Ecca, Beaufort, Stormberg Groups and mainly consist of interbedded conglomerate, sandstone, siltstone, and claystone (Fig. 1D). They are then intruded by Jurassic and Cretaceous dykes and sills. The Cenozoic stratigraphic succession cover is poorly studied but several calcrete levels are described^27^.

## Methods

**Figure 2:**
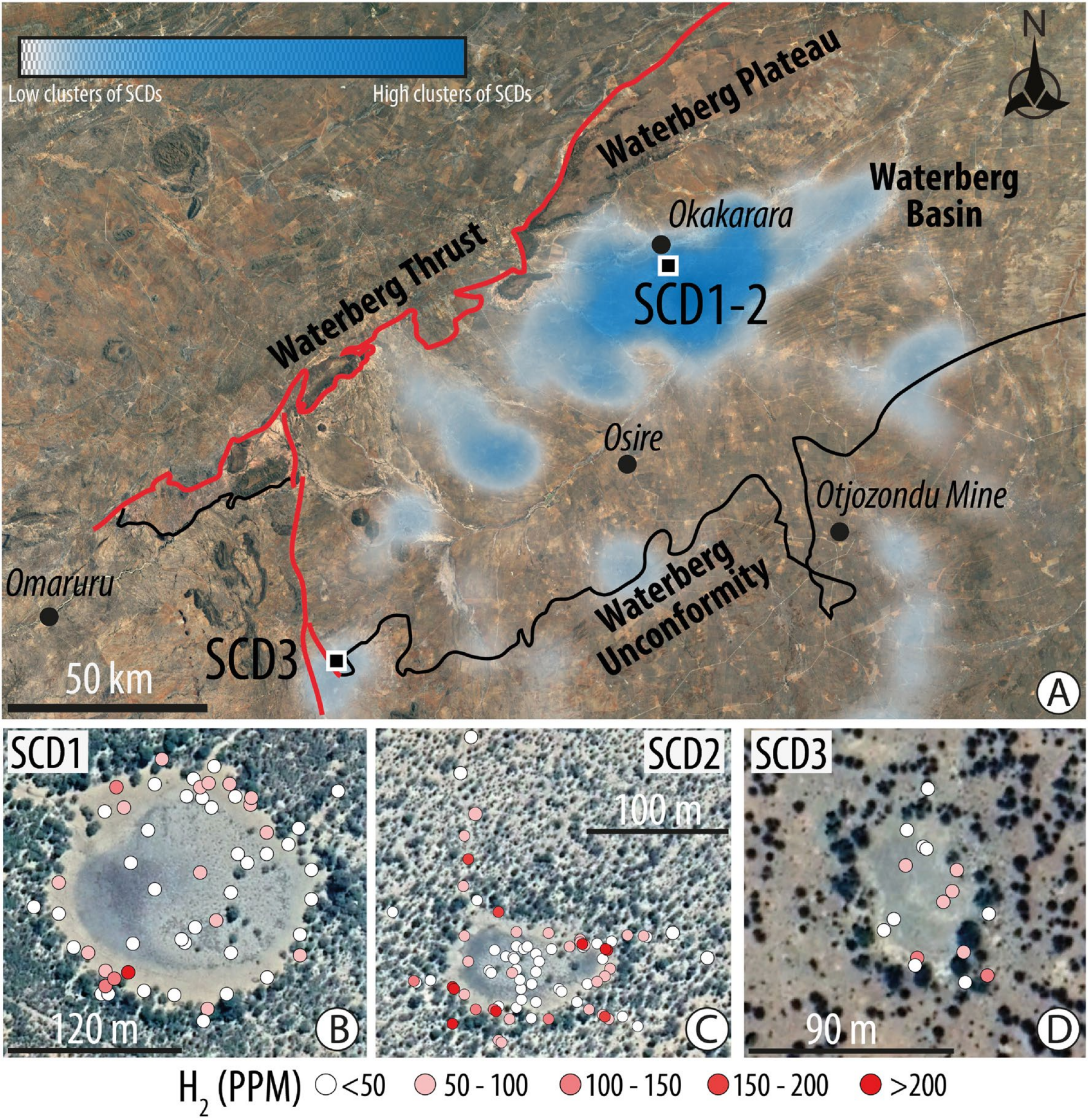
H_2_ seeps in Waterberg Basin. (**A**) Density map showing the spatial distribution of the SCDs within Waterberg Basin. The Heatmap plugin uses kernel density estimates and was created using the Free and Open Source QGIS (QGIS.org, %Y. QGIS Geographic Information System. QGIS Association. http://www.qgis.org). More than 2200 SCDs were identified by remote sensing techniques and mapped according to the recognition criterion defined in Lévy et al.^5^. (**B**–**D**) SCDs onto which the BIOGAS 5000 results of H_2_ concentration are overlain. SCD1 (**B**) (location: − 20.651°/17.468°); SCD2 (**C**) (location: − 20.627°/17.466°); SCD3 (**D**) (location: − 21.527°/16.633°). Note that H_2_ concentrations are lower in the center of the structure and higher at its periphery, in line with previous studies^6,7^.

**Figure 3:**
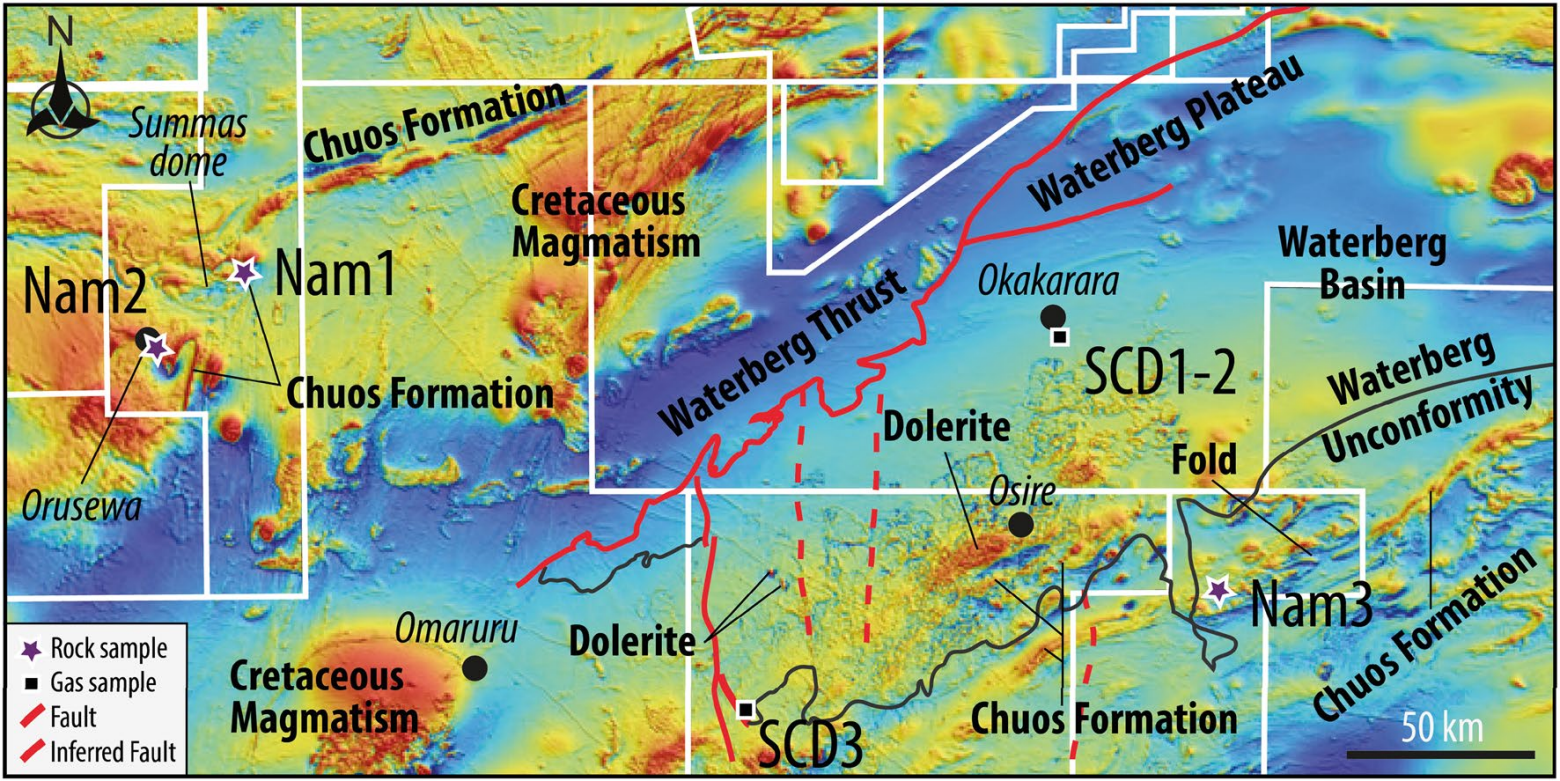
Magnetic data and geology of Namibia. Colors shaded Total Magnetic Intensity map from Hutchins and Wackerle (Ref.^30^). The data is a compilation of high-resolution and regional magnetic data. The white lines correspond to the outlines of the high-resolution airborne magnetic/radiometric surveys. Lithologies and faults are identified thanks to the geological maps from the Geological Survey. Note that red stands for positive magnetic anomaly while blue stands for the opposite.

We investigated whether Neoproterozoic banded iron formations could generate H_2_ as a result of interactions with Waterberg Basin hydrogeological systems and formation waters. We first used multispectral analyses to identify SCDs in the basin, followed by soil gas measurements. In the field, we used the BIOGAS 5000 instrument which also measures the composition of O_2_, CO_2_, CH_4_, and H_2_S gas concentrations in the bulk gas. In situ gas measurements were conducted on three SCDs during three different fieldworks in Waterberg Basin. Data already existed on two of them, SCD1 and SCD2^6^. Additionally, we collected and analyzed a few samples using gas chromatography (GC) in the laboratory. For more information on the sampling method, please refer to Ref.^5^. Results are presented in Fig. 2 and Supplementary Material (Tables S1 and S2). We then investigated the H_2_-generating potential of Chuos Formation because it is a major iron-rich formation within Damara Belt stratigraphy. We therefore collected and analyzed three representative rock samples (Nam1, Nam2, and Nam3) of the Chuos Formation distributed in the surroundings of the basin: one from the area of Summas dome (Nam1), a second one from the area of Orusewa (Nam2), and a last one sampled in the drillcore from the Otjozondu mine (Nam3) (Fig. 3). Importantly, more information on iron ore for Nam2 and Nam3 is detailed in Ref.^22^ and Ref.^28^, respectively. Lithologies related to Nam1 and Nam2 underwent low-*T* and low-*P* whereas Nam3 recorded high-*T* medium-*P* conditions according to the metamorphic map from Ref.^13^. We determined the iron content and speciation in the rock samples subsequently, using Inductively Coupled Plasma Atomic Emission Spectrometer (ICP AES) and titration in solution to estimate the H_2_-generating potential of Chuos Formation. X-ray diffraction analyses were also performed to evaluate the mineralogy of the bulk rock samples. Methods are detailed in Supplementary Material. Finally, we considered different lithologies of Waterberg Basin, evidence, and characteristics of fluid circulation and accumulation.

## Results

### H_2_ seepages from Waterberg Basin

Figure 2A presents the spatial distribution of SCDs per surface area unit, and indicates several areas of interest in Waterberg Basin. The highest density of SCDs occurs in the central basin a few kilometers away from Waterberg Plateau. Several areas with a high density of SCDs are also present close to the contact, a major unconformity, between Neoproterozoic metamorphic units and the younger sedimentary rocks. Overall, however, the SCDs distribution appears to be random. H_2_ measurements were performed on samples from three SCDs, and results are shown in Fig. 2B–D. Concentrations of H_2_ obtained using the BIOGAS 5000 field instrument ranged from 0 to 395 ppm (Table S1). GC results yield concentrations of H_2_ ranging from 1 to 730 (Table S2). These results are consistent with the BIOGAS 5000 data. In addition, no logic between the different gases has been established (Supplementary Material, Fig. S1).

### Metamorphosed Chuos Formation: from the regional to the sample-scale

Chuos Formation outcrops in the Northern and Central Zones (Fig. 1A). Figure 3 presents the magnetic response map of the different lithologies around Waterberg Basin, obtained from the Geological Survey of Namibia. Interestingly, the geological maps from the survey of Namibia show that the most significant anomalies correspond to Chuos Formation and magmatic activities (mostly Cretaceous and Jurassic in age). Chuos Formation crops out around Waterberg Basin, to the northwest (Fig. 3), to the west in the Navachab mine, and to the south in the Otjozondu mine (Figs. 1 and 3). At some places, the maps predict Chuos Formation to continuously outcrops over several kilometers with an estimated thickness of several hundreds of meters, which is consistent with our field observations. Considering its distribution, it is inferred to underlie much if not most of Waterberg Basin. Wherever Chuos Formation is deformed and metamorphosed as evidenced by the presence of biotite. The foliation is generally sub-vertical with a variable strike, while repeated sedimentary sequences indicate the presence of kilometer-scale isoclinal folds, also identifiable on the Total Magnetic Intensity map (Fig. 3). The area of Osire, which is located at the central part of Waterberg Basin, exhibit a more irregular and variable magnetic signal intensity. There, several high-intensity signals oriented NE-SW are identified and could correspond to Chuos Formation within the basement below the basin. Magmatic intrusions may also be present locally (see the oval shape located SW of Osire, Fig. 3). Such interpretation is consistent with the study of Christelis and Struckmeier^27^ that reported the presence of intrusive dolerites in this area. In summary, the presence of Chuos Formation under the basin is strongly suspected. Additional support is provided by other geophysics data that indicate a shallow crustal conductor in the upper to mid-lower crust, in the region of Waterberg Basin^29^. This latter may be related to the presence of oxide-rich lithologies, such as magnetite in banded iron formations, and sulfides^29^.

**Figure 4:**
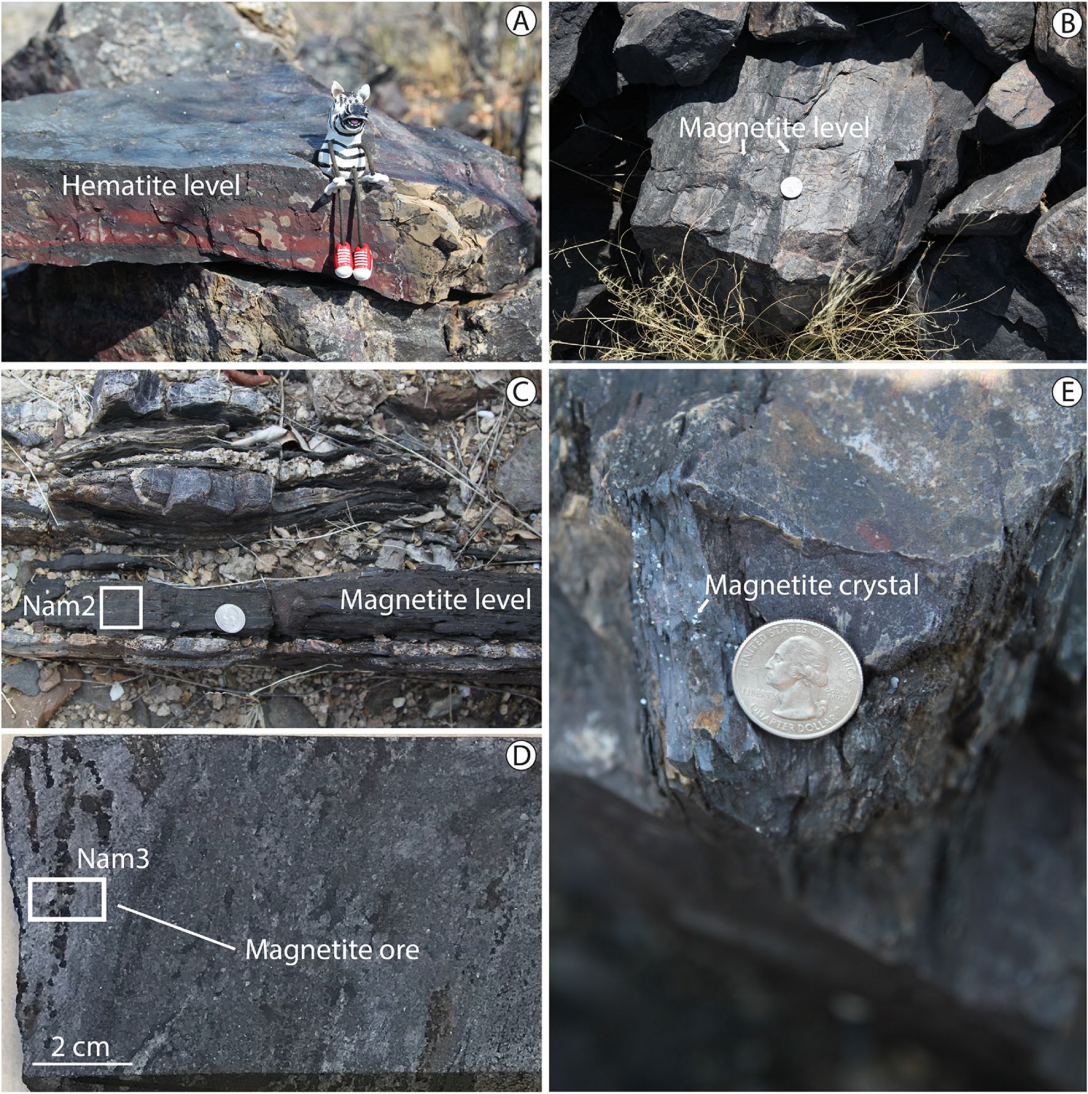
Field pictures showing ironstones. (**A**) Banded hematite level (location: − 20.30°/15.43°). Foliation strikes NNE-SSW and dips 65°. (**B**) Block of rock belonging to a 50-m thick succession of iron-rich lithologies (S 70/60). The dark layers contain magnetite (location: − 20.67°/15.14°). (**C**) Sub-vertical magnetite centimetric level between the dark diamictites (location: − 20.49°/15.34°). Nam2 corresponds to the sample 2. (**D**) Core sample from the Otjozondu manganese mine (location: − 21.23°/18.04°). The right part of the core sample is less enriched in magnetite. Nam3 corresponds to the sample 3. (**E**) Crystals of magnetite in fracture plane (location: − 20.30°/15.43°).

In the field, Chuos Formation is mainly composed of interbedded diamictites and cherty iron beds that are the two dominant sedimentary facies: one siliceous and the other carbonate. Both facies are not laterally continuous due to deformation. Each can be several meters in thickness. Similar to previous studies (e.g.^20,22^), these iron beds exhibit an unusual scarcity of clastic detritus. Iron oxides are observed in diamictites but higher concentrations are found in banded iron formations. In these layers, although hematite is usually present (Fig. 4A), magnetite is predominant in some samples and various habits (Fig. 4B–E) including:

(i) Disseminated magnetite, 10–100 µm grains (Fig. 4B), roughly subhedral, sometimes organized into thin to very thin beds about c. 1–5 cm;
(ii) Fine-grained magnetite, about 5–10 µm grains, in laminations and very thin bed to a few cm thick (Fig. 4C,D) parallel to the main foliation;
(iii) Porphyroblastic magnetite in fractures and around the quartz veins (Fig. 4E).

**Figure 5:**
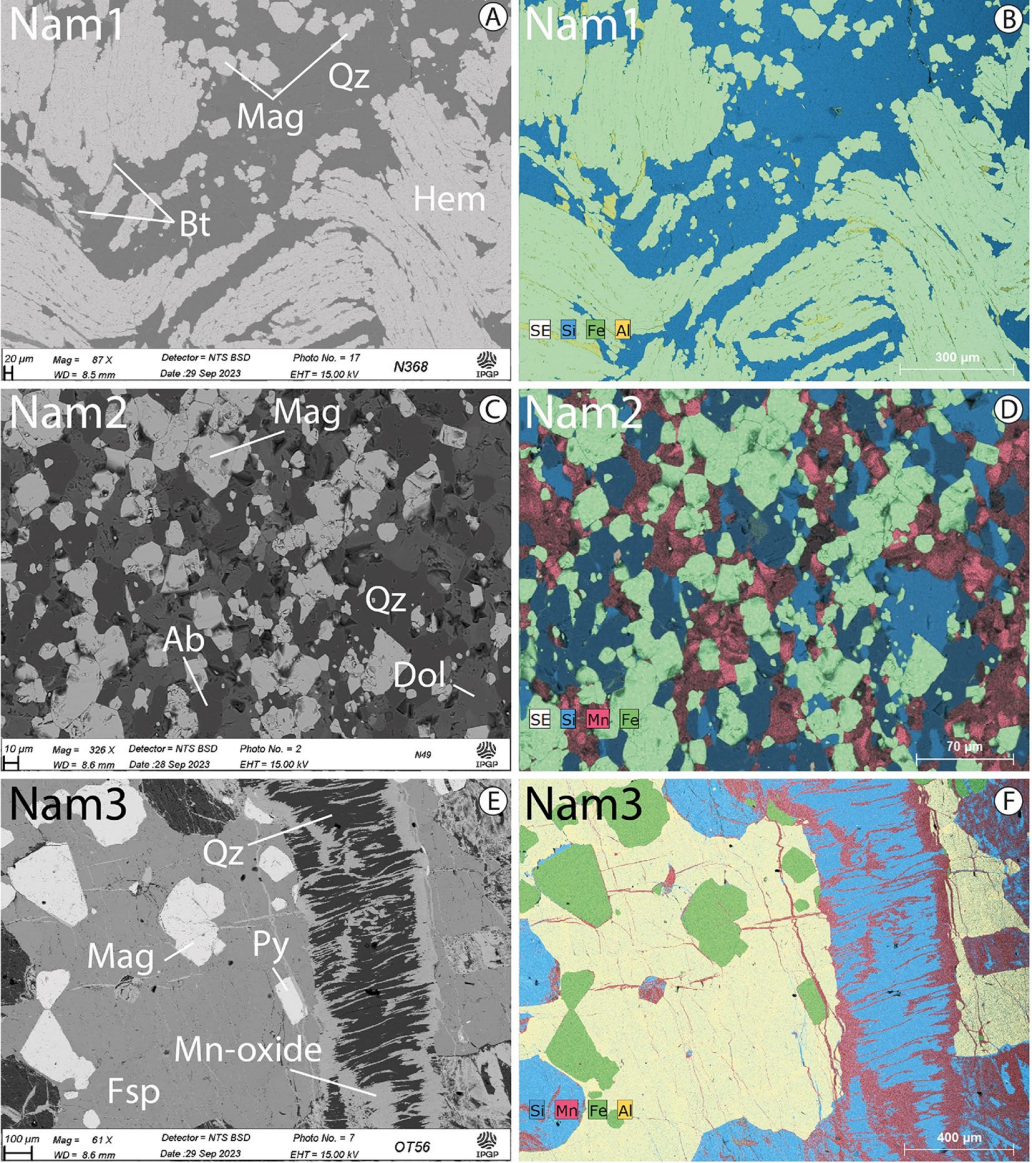
Back-scattered electron images and related elementary mapping of the three representative rock samples. (**A**,**B**) Paragenesis of sample Nam1. Hematite (Hem) ore comprising layers of densely packed hematite elongated and folded whereas magnetite (Mag) comprising small aggregated anhedral crystals. Biotite (Bt) contains also Fe^2+^—sample Nam1 (location: − 20.46°/15.27°). Quartz (Qz). (**C**,**D**) sample Nam2 is mainly composed of octahedral and anhedral crystals of magnetite. Other minerals present are albite (Ab), dolomite (Dol), and quartz. Note that dolomite is Fe^2+^-enriched according to our ICP-AES and the titration results—sample Nam2 (location: − 20.49°/15.34°). (**E**,**F**) sample Nam3 is from the Otjozondu manganese mine and consists of randomly oriented porphyroblasts Mn-rich magnetite, quartz, feldspars (Fsp), and pyrite (Py). Such an assemblage is crosscut by quartz veins bearing Mn-oxide precipitates—sample Nam3 (location: − 21.23°/18.04°).

Fe_2_O_3_ total ranges from 50 to 70 wt% and Fe^2+^ ranges between 3.4 and 12.5 wt% (Table 1). Mineralogy indicates sample Nam1 consists of quartz, hematite ± magnetite ± Fe-rich biotite (Fig. 5A,B, S2A), sample Nam2 is composed of quartz, Fe-rich dolomite, albite, magnetite ± Fe-rich biotite (Fig. 5C,D, S2B). Whereas sample Nam3 is composed mainly of quartz, porphyroblastic magnetite, anorthite ± baryte (Fig. 5E,F, S2C). The detailed samples mineralogy of the samples is shown in Supplementary Material (Table S3). Interestingly, accessory minerals such as dolomite and biotite contain also Fe^2+^. Overall, our results are consistent with previous studies in which numerous sample analyses were acquired (see Ref.^22^ for Nam2 and Ref.^28^ for Nam3).

**Table 1:** Major element and titration data from our samples.

Samples	SiO_2_%	TiO_2_%	Al_2_O_3_%	FE_2_O_3_(T)%	MnO%	MgO%	CaO%	Na_2_O%	K_2_O%	P_2_O_5_%	LOI%	Total%	Fe^2+^(Wt%)	Fe_2_O_3_ (Wt%)
Nam1	*19.26*	*0.06*	*0.67*	*73.02*	*0.01*	*0.2*	*0.05*	*0.1*	*0.3*	*0.13*	*0.02*	*93.83*	**3.43**	**62.28**
Nam1	*15.21*	*0.07*	*0.67*	*75.65*	*0.01*	*0.25*	*0.02*	*0.13*	*0.26*	*0.14*	*0.02*	*92.43*	**3.62**	**62.09**
Nam2	*21.9*	*0.19*	*3*	*49.6*	*2.02*	*2.81*	*8*	*1.49*	*0.1*	*0.41*	*9.43*	*98.89*	**12.48**	**44.65**
Nam2	*22.52*	*0.18*	*2.83*	*50.28*	*2.01*	*2.81*	*8.12*	*1.59*	*0.16*	*0.41*	*9.43*	*100.44*	**12.47**	**44.68**
Nam3	*8.56*	*0.38*	*0.85*	*57.76*	*20.28*	*0.41*	*0.42*	< LD	*0.05*	*0.14*	*0*	*88.85*	**4.73**	**47.24**
Nam3	*7.27*	*0.37*	*0.84*	*60.4*	*18.52*	*0.44*	*0.4*	*0.04*	*0.03*	*0.15*	*0*	*88.46*	**4.68**	**47.3**

The table indicates the bulk rock major elements analyzed by ICP AES in italic and the titration results in bold. The analyses for each sample were duplicated.

### An inferred H_2_ system: lithologies of interest for fluid circulation and accumulation

**Figure 6:**
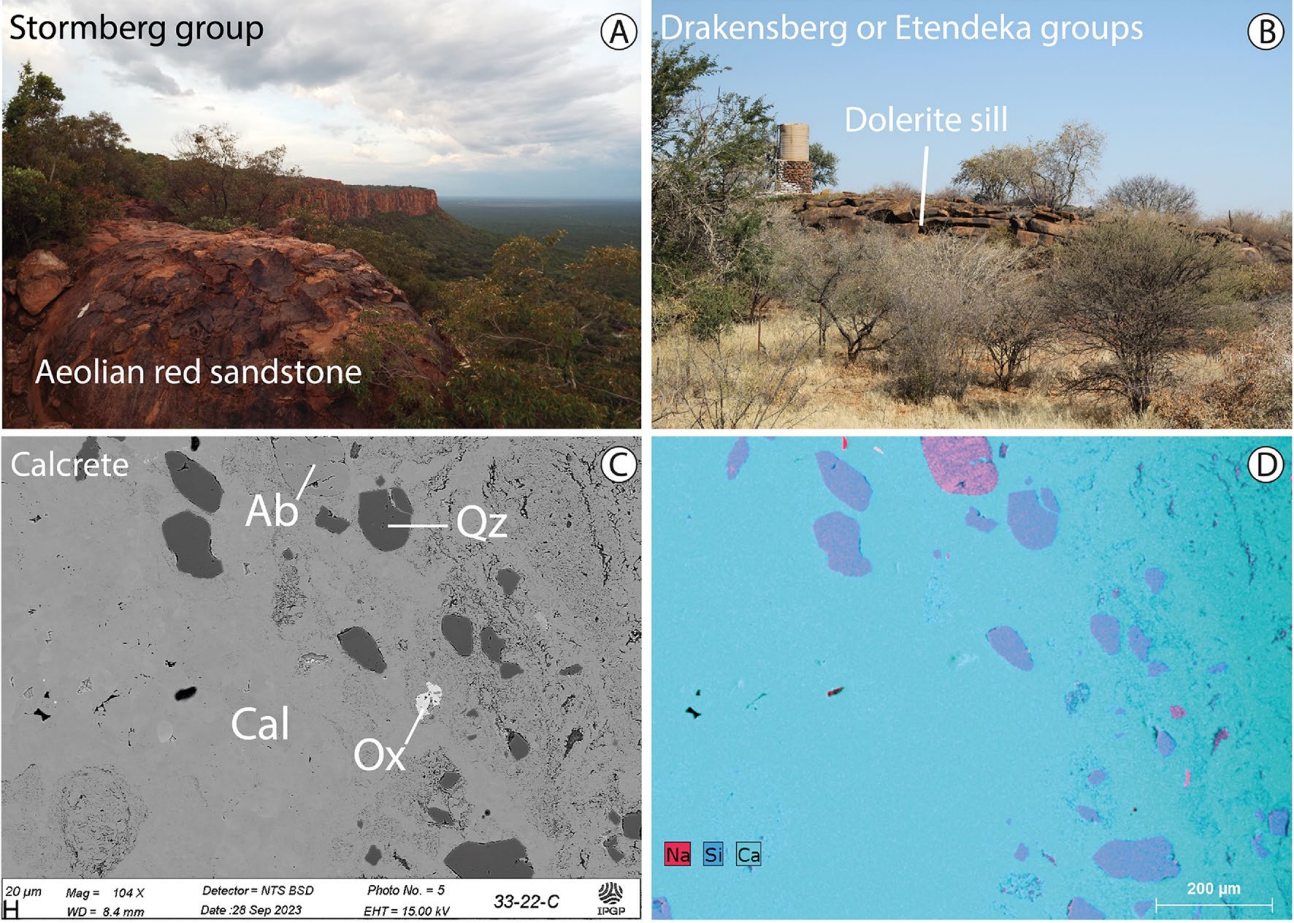
Main lithologies of interest for a inferred H_2_ system. (**A**) Aeolian sandstones defining Waterberg Plateau (location: − 20.51°/17.24°). (**B**) Sill of dolerite outcropping at the edge of the basin (location: − 21.51°/16.58°). (**C**,**D**) Example calcrete sample observed in Waterberg Basin. Paragenesis is composed of calcite (Cal) including clasts of Ab and Qz. Reflected light image (**C)** and related elemental mapping (**D**).

The flat-lying Stormberg Group aeolian sandstone surfaces from the Karoo Sequence form Waterberg Plateau (Figs. 1D and 6A). These are highly porous and have the proper characteristics to permit water infiltration because of their high porosity^27^. Indeed, several artesian springs are located on the southern slope of Waterberg Plateau and the northern edge of the Klein Waterberg.

Underlying the aeolian sandstone, the Omingonde Fm., mudstones, sandstones, and conglomerates, is locally up to 510 m in thickness^26,31^. Although this unit has a low groundwater potential due to its clay content (i.e., acting as an aquiclude), Christelis and Struckmeier^27^ reported water in this formation near Osire town (Fig. 3) and confirmed that contact zones of intrusive dolerites may be much better aquifers. Thus, this unit too may be considered as a potential aquifer and the dolerite sills present in this region may act as seals for H_2_ reservoirs, as observed in association with Mali hydrogen-producing wells^1,2^. At some localities, the dolerite is defined by a flat-lying surface (S 75/08) reaching several meters of thickness (Fig. 6B). These undated but probably Jurassic or Cretaceous intrusions are described on the geological map, intruding the basement and the sedimentary rock succession at the edge of the basin, south and east of Waterberg Plateau. These intrusions are also drilled SE of Grootfontein (Borehole ROD-37, 100 km NE of Otjikoto mine) suggesting they likely occur over a large part of the basin.

Calcrete is another lithology that may also act as a fluid barrier (Fig. 6C,D). Formed by solution and redeposition of calcium carbonate, such a unit consists of hard cement covering a large area in Waterberg Basin and northward around the Otjikoto mine (Fig. 1A). Christelis and Struckmeier^27^ reported that calcrete layers formed by fluvial process in Namibia may reach 100 m in thickness and occur at depths of up to 150 m below the surface.

## Discussion

### The metamorphosed Chuos Formation: a potential source of natural H_2_ in Waterberg Basin

In the context of natural H_2_ exploration, the generating potential of Archean banded iron formations was recently investigated since their Fe^2+^ content is very high^9^. They usually consist in Fe^2+^-bearing minerals that are accepted to source H_2_ during water–rock interactions such as magnetite (α − Fe_3_O_4_) or siderite (FeCO_3_), according to the equations Eqs. (1), (2)^10,32^:

1 $${2}\left( {\upalpha {-}{\text{Fe}}_{{3}} {\text{O}}_{{4}} } \right)\, + \,{\text{H}}_{{2}} {\text{O}} \to {3}\left( {\gamma \, - \,{\text{Fe}}_{{2}} {\text{O}}_{{3}} } \right)\, + \,{\text{H}}_{{2}}$$

2 $${\text{3FeCO}}_{{3}} \, + \,{\text{H}}_{{2}} {\text{O}} \to \left( {\upalpha {-}{\text{Fe}}_{{3}} {\text{O}}_{{4}} } \right)\, + \,{\text{H}}_{{2}} \, + \,{\text{3CO}}_{{2}}$$

On the contrary, the H_2_ potential of Neoproterozoic banded iron formations has been considered very limited up to now, since iron is expected to occur predominantly as Fe^3+^ in such lithology. Interestingly, our analyses reveal the presence of Fe^2+^ in the metamorphosed Chuos Formation, mainly found in magnetite and locally in other minerals. This implies that metamorphism seems to favor higher Fe^2+^ concentrations, which are preserved in different ways compared to unmetamorphosed Chuos Formation where the amount of Fe^2+^ is low (magnetite is rare and appears isolated,^21^. Interestingly, based on forty-six samples, Lechte et al.^22^ proposed similar conclusion indicating that iron in the metamorphosed Chuos Formation is mostly present as magnetite. Thus metamorphosed Neoproterozoic banded iron formations contain Fe^2+^, which may, like Archean banded iron formations be sites of H_2_ generation^9^, through similar reaction pathways (see Eqs. 1 and 2). Nonetheless, our data show that Fe^2+^ quantities vary depending on location, which will strongly influence the amount of H_2_ produced.

Further, a recent synthesis of available experimental data^5^ showed that redox reactions involving magnetite seem to promote H_2_ generation better than other processes such as serpentinization at near-ambient temperatures. Therefore, we recommend that magnetite oxidation be studied in more detail in continental settings, especially where banded iron formations are present. Furthermore, serpentinization also produces magnetite that can, in turn, be altered, and result in a second, augmentation of H_2_ generation at lower temperatures. Thus, it is reasonable to inquire into the geological origins of natural H_2_, especially as a function of different combinations of geochemical processes at various pressure and temperature conditions.

**Figure 7:**
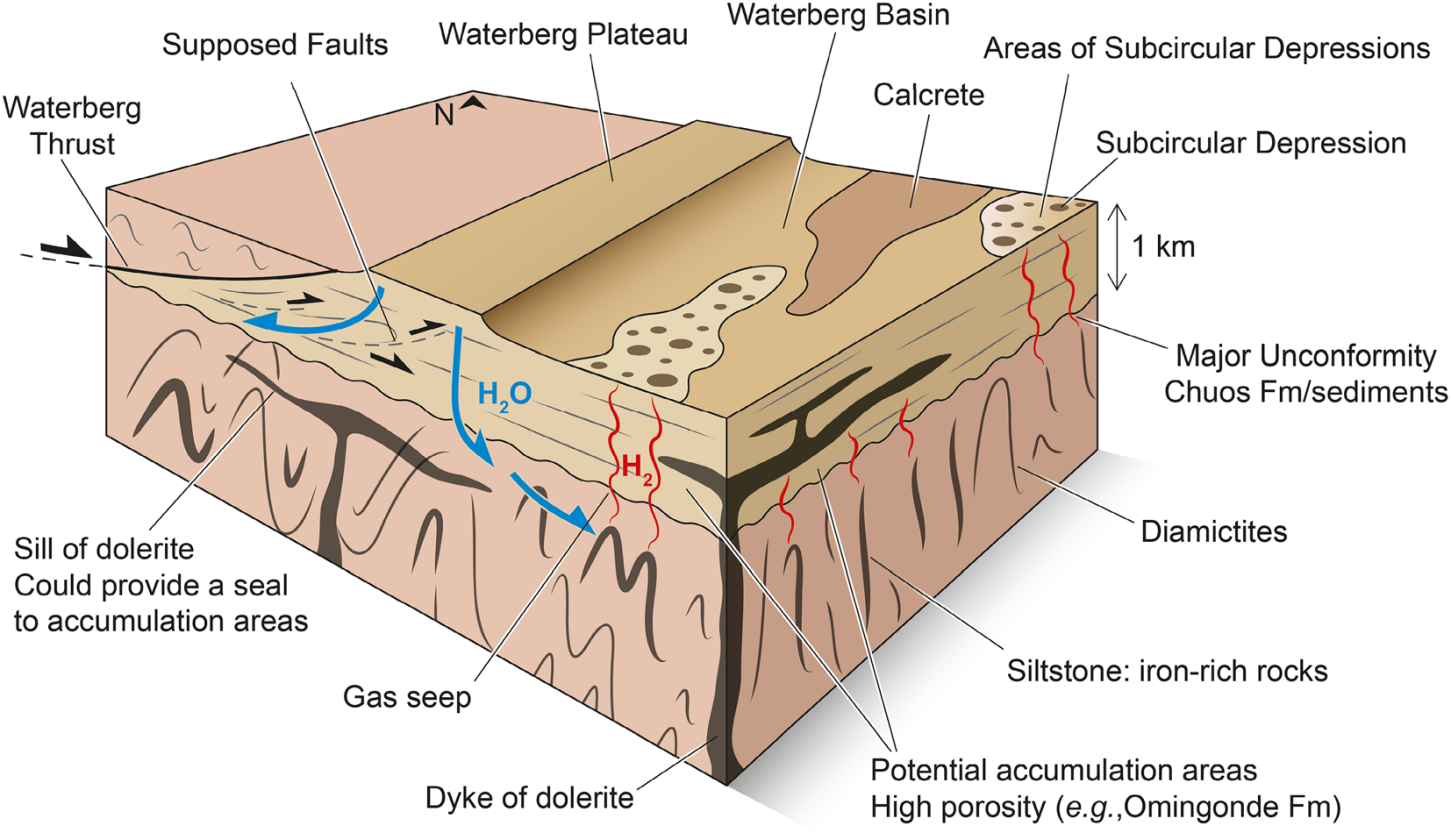
Conceptual model of the inferred H_2_ system. Waterberg fault is indicated by a thick black line and other faults by a thin black dotted line that corresponds to faults from the study of Granath et al.^26^. Unfortunately, there is no seismic data in the basin so the position and the role of faults on fluid circulation is unknown.

As previously shown, the metamorphosed Chuos Formation is present throughout the region in large quantities. We suggest therefore that this large sedimentary iron resource available could therefore generate a potentially significant amount of H_2_. The presence of thousands of SCDs distributed widely across the whole Waterberg Basin and their association with H_2_ seeps supports this inference (Figs. 2 and 7). In detail, we propose that magnetite gets partially converted at depth into maghemite (γ-Fe_2_O_3_), a metastable Fe-oxide only containing Fe^3+^, before reaching its stable state corresponding to hematite. Importantly, such a reaction has only been tested successfully at T ≤ 200 °C up to now^10^. In addition, the destabilization of other Fe^2+^-rich minerals (e.g. Fe^2+^-biotite, -carbonates) may also lead to H_2_ generation, and probably at different depths and *P*–*T* conditions. Further, several factors should also be considered to better characterize the H_2_-generating “kitchen”. In particular, the porosity of Fe-bearing rocks as well as the nature of the minerals present (e.g. size, geometry) should be further investigated in the future since they are assumed to strongly impact reaction kinetics.

### H_2_ system in Namibia

After identifying a possible source rock, water appears to be the second key factor controlling the generation of H_2_. In Waterberg Basin, two assumptions can be discussed concerning the origin of water supply at depth, with direct consequences on the dynamics of the inferred H_2_ system. On the one hand, the water could be hydrothermal, related to igneous magmatic activity which is widespread in the study area (Fig. 3). This implies that the H_2_ was generated contemporary to magmatism emplacement. We could therefore currently observe surface seeps from a long-term H_2_ system over a large area of Waterberg Basin. Such a long-term accumulation implies an efficient combination of reservoir-seal. There, a major question arises: how to explain the formation of seeps? In other words, seeps (i) may have existed since the emplacement of magmatism to the present day, involving huge quantities of H_2_ lost, or (ii) may have developed more recently due to, for example, a tectonic activity that partially destabilized the reservoir at depth. Addressing such a question may help find an explanation for the spatial distribution of SCDs and faults in Waterberg Basin. Despite the absence of seismic data, this doesn't seem to be the case because the SCDs distribution is random (Fig. 2A). On the other hand, water supply could originate from meteoric water infiltration. Infiltration could be facilitated by regional faults at the margins of Waterberg Basin. An active hydrogeological circulation is indicated by the topography of Waterberg Plateau, the highly porous aeolian sandstones that cap the plateau, and the presence of springs at the foot of the hill (Fig. 2A). In that case, the inferred H_2_ system may be considered as dynamic, with no trap mandatory although a seal could impede the flow and facilitate accumulations^33^. Where water reaches the major unconformity (Fig. 7), it can then infiltrate into the fractured metamorphic basement and leach rocks. This provides opportunities for the oxidation of magnetite caused by water reduction as well as the destabilization of other Fe^2+^-rich minerals (e.g. Fe^2+^-biotite, -carbonates) of Chuos Formation.

Once generated, H_2_ migrates and reaches the surface in free form with other gases (e.g. CH_4,_ CO_2_—see Tables S1 and S2 in Supplementary Material). Unfortunately, there is currently no constraint on the spatial relationships between where H_2_ forms, where it might be accumulated, and/or how it migrates toward seepage sites. Is it in the form of a free gas separate phase, in aqueous solution, or diffused? Several relevant points can be suggested for future works:

(i) The absence of a clear alignment of SCDs implies that the porous nature of sediments forms primary controls on vertical gas pathways toward the surface even though there seems to be a link between the SCD3 and the basin boundary fault;
(ii) The size of the area affected by H_2_ seeps suggests that a carrier bed may be present below the surface. As previously mentioned, the Omingonde Fm. is a geological reservoir in particular cases, where H_2_ in a dissolved phase could circulate, before being locally returned to a gaseous phase depending on several factors (e.g. temperature, pressure);
(iii) The magmatic doleritic sills present in the basin as well as calcrete may in parallel act as seals to allow accumulation of H_2_ (Fig. 7).

## Conclusion

To conclude, magnetite-bearing Neoproterozoic sedimentary rocks are lithologies that can potentially support the generation of H_2_ on continents even though alternatives cannot be excluded (e.g. radiolysis). This studied portion of Namibia exhibits the major geological criteria, the most important of which are active hydrogen-rich gas seeps, that, using the analogy of petroleum systems, suggest Waterberg Basin is a prospective site for the accumulation of natural hydrogen gas that potentially could be produced in the future if further exploration efforts are successful. Finally, banded iron formations that constitute more than 60% of global iron ore reserves, should be targeted. Further investigation is necessary to test the significance of banded iron formations for H_2_ seepage as well as to understand the associated reactions and kinetics.

### Supplementary Information

Supplementary Information 1.

Supplementary Table S1.

Supplementary Table S2.

Supplementary Table S3.

## Data Availability

All data generated or analyzed during this study are included in this published article and its supplementary information files.
