# Supplementary material for: A new continental hydrogen play in Damara Belt (Namibia)
Source: Sci Rep. 2024 May 22;14:11655. doi: 10.1038/s41598-024-62538-6 (PMC11111756; doi:10.1038/s41598-024-62538-6)
Supplement: Supplementary file 1 — Supplementary Information 1. [file 41598_2024_62538_MOESM1_ESM.docx]

**A New Continental Hydrogen Play in Damara Belt (Namibia)**

V. Roche^1,2∗^, U. Geymond^3^, M. Boka-Mene^4^, N. Delcourt^4^, E. Portier^4^, S. Revillon^5^ and I. Moretti^1^

^1^Laboratoire des Fluides Complexes et leurs Réservoirs - IPRA, E2S-UPPA, TotalEnergies, CNRS, Université de Pau et des Pays de l'Adour, UMR5150 Pau, France

^2^Laboratoire de Planétologie et Géosciences, LPG UMR 6112, CNRS, Le Mans Université, Univ Angers, Nantes Université, Avenue Olivier Messiaen, 72085 - Le Mans, France

^3^ IPGP, Paris, France

^4^45-8 Energy, Lyon, France

^5^﻿SEDISOR/LGO UMR, Plouzané, France

# **SUPPLEMENTAL DATA**

# **Methods**

The bulk rock major elements were analyzed by Inductively Coupled Plasma Atomic Emission Spectrometer (ICP AES) Horiba Jobin Yvon Ultima 2 at the Pôle de Spectrométrie Océan (PSO/IUEM, Plouzané, France), following the protocol adapted from Cotten *et al*. (ref 2). Each powdered sample was digested in Teflon vials with HF 32N and HNO_3_ 14.4N and the dry residue was dissolved in a H_3_BO_3_ solution. Mica-Fe and IF-G international standards were used as internal and external control. The precision of measurements performed on that instrument is usually better than 4% for concentrations above 1%. FeO (wt%) was determined by KMnO_4_ titration after a HF + H_2_SO_4_ partial dissolution following a method modified from Jen (ref 3) and Teagle (ref 4).

XRD analyses were performed on a Malvern-Panalytical Empyrean diffractometer equipped with a copper tube (Kα = 1.541874 Å) and a Malvern-Panalytical multi-channel PIXcel detector (UPC, Paris). The chosen angular range was between 5° and 80°, the step size was 0.007°, and the time per step was 80 s (*i.e.*, 1 h measurement per sample). Diffractograms were then interpreted using Highscore Plus software from Malvern-Panalytical.

**Gas results**

Table S1: Table shows the raw gas compositions of the gases measured on the field.

Table S2: Table shows the raw gas compositions of the GC gases measured in the laboratory.

**
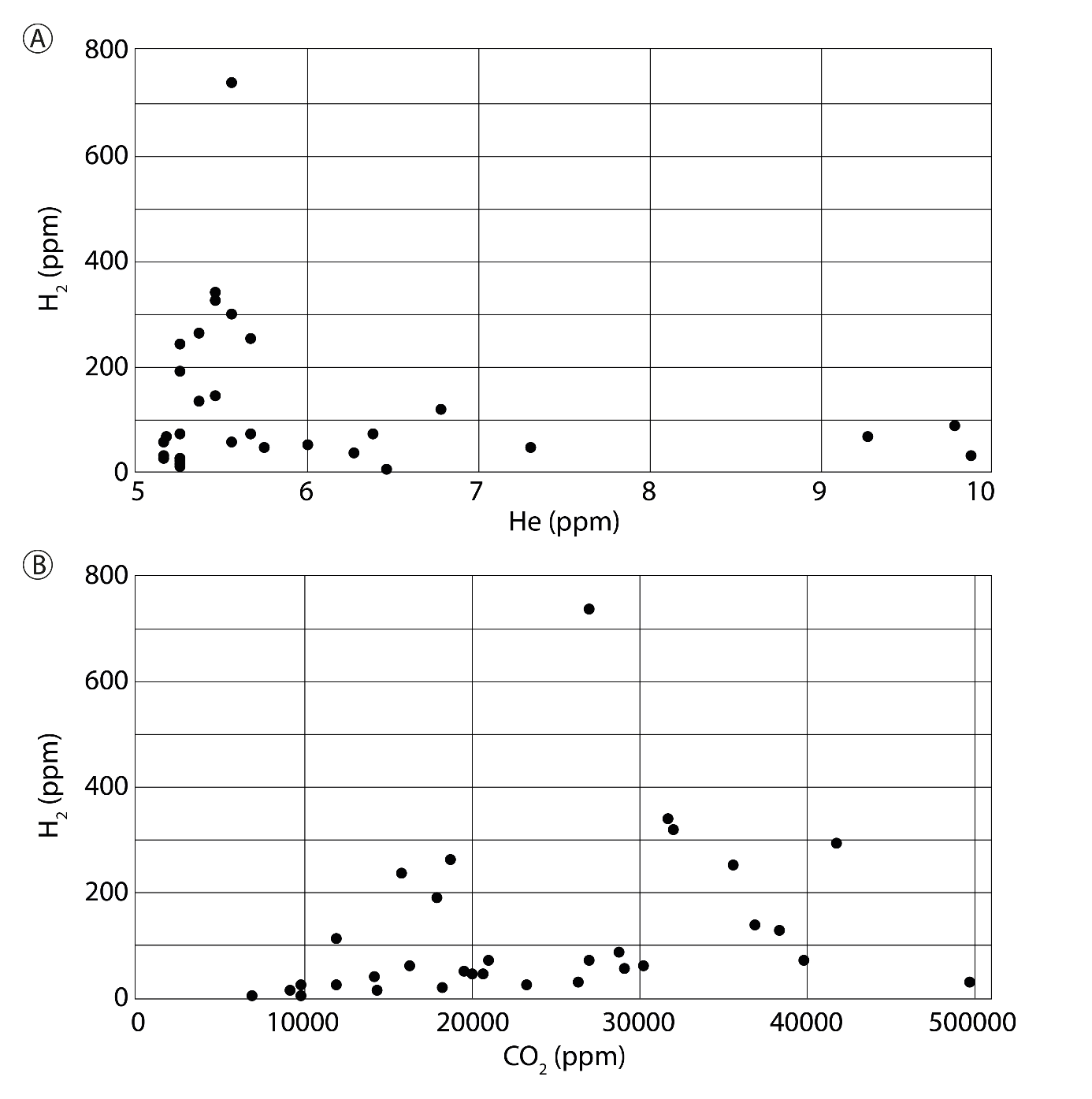
**

**Fig. S1: GC results.** Graphs showing a lack of relationship between (**A**) H_2_ and He and (**B**) H_2_ and CO_2_.

**Rock results**


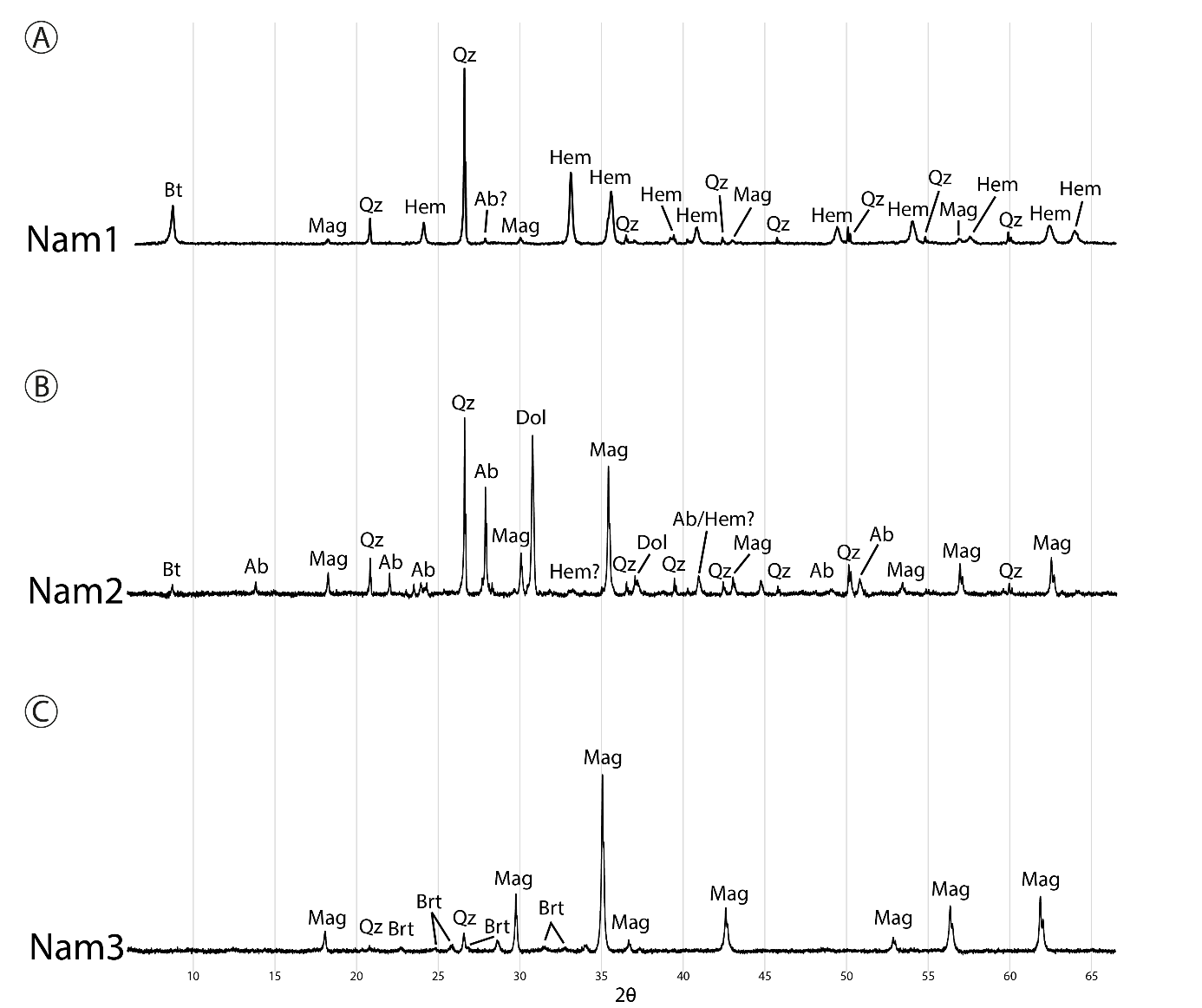


**Fig. S2: XRD results. A-C,** XRD results of sample Nam1 (**A**), sample Nam2 (**B**) and sample Nam3 (**C**). Note that the magnetite peaks are slightly left-shifted in sample Nam3 since magnetite incorporates manganese: Jacobsite (Fe,Mn)Fe^3+^_2_O_4_ (see Figs. **5E-F**). Abbreviations: Albite (Ab), Biotite (Bt), Baryte (Brt), Dolomite (Dol), Hematite (Hem), Magnetite (Mag), Quartz (Qz).

**Table S3:** Table showing the main minerals from our samples based on thin section observations, XRD data, and bulk rocks analysis.

*References*

1. Lévy, D., Roche, V., Pasquet, G., Combaudon, V., Geymond, U., Loiseau, K., Moretti^,^ I. (2023). Natural H_2_ exploration: tools and workflows to characterize a play. STET
2. Cotten, J., A. Le Dez, M. Bau, R.Maury, P. Dulsky, S. Fourcade, M. Bohn, and R. Brousse (1995), Origin and anomalous rare-earth element and yttrium enrichments in subaerially exposed basalts: Evidence from French Polynesia, Chem. Geol., 119, 115–138, doi:10.1016/0009-2541(94)00102-E.
3. Jen, L.S. (1973) The determination of iron(II) in silicate rocks and minerals, Analytica Chimica Acta, Volume 66, Issue 2.
4. Teagle, D.A.H. (1993) A study of hydrothermal alteration of the Troodos ophiolite, Cyprus: petrological, geochemical and strontium isotopic constraints. PhD thesis, University of Cambridge.
